# The Mycetophagidae (Coleoptera) of the Maritime Provinces of Canada

**DOI:** 10.3897/zookeys.64.553

**Published:** 2010-10-22

**Authors:** Christopher G. Majka

**Affiliations:** >Research Associate, Nova Scotia Museum, 1747 Summer Street, Halifax, Nova Scotia, Canada

**Keywords:** Coleoptera, Mycetophagidae, Mycetophaginae, *Mycetophagus*, *Litargus*, *Typhaea*, aritime Provinces, Canada, biodiversity, fungus beetles, rare species

## Abstract

The Mycetophagidae (hairy fungus beetles) of the Maritime Provinces of Canada are surveyed. Seven species in the genera Mycetophagus, Litargus, and Typhaea are found in the region. Six new provincial records are reported including Mycetophagus punctatus and Mycetophagus flexuosus, whichare newly recorded in the Maritime Provinces. The distribution of all species is mapped, colour habitus photographs of all species are figured, and an identification key to species is provided. The discussion notes that four of the species found in the region are apparently rare, possibly due to the history of forest management practices in the region; a situation similar to that of a significant proportion of other saproxylic beetles found in the Maritime Provinces.

## Introduction

The Mycetophagidae (hairy fungus beetles) are a family of relatively small, fungus-eating beetles. Only five genera and 26 species are known in North America, 15 of which have been recorded in Canada (Bousquet 1991; Young 2002). Parsons (1975) provided the most recent species-level taxonomic revision of the family. Only two species, Mycetophagus quadriguttatus Müller and Typhaea stercorea (Linnaeus), have previously been recorded from the Maritime Provinces of Canada (New Brunswick, Nova Scotia, and Prince Edward Island) (Bousquet 1991). Species of Mycetophagus are commonly found in the decaying fruiting bodies of mushrooms and fleshy polypores, particularly those that have begun to dehydrate (Young 2002). Typhaea stercorea (Linnaeus), an adventive Palaearctic species, is associated with a large variety of moldy substances and is found both outdoors in natural environments, and indoors in association with a variety of stored products (Campbell et al. 1989). The biology of Litargus species is poorly known, however, there are records of a number of species associated with fungi, under bark, and in decaying logs (Schwarz 1876; Cline and Leschen 2005; Ulyshen and Hanula 2010). The present study reports the results of an investigation into the biodiversity of this family in the Maritime Provinces.

## Methods and conventions

Acronyms (largely following Evenhuis 2010) of collections referred to in the text are:

ACNSAgriculture and Agri-Food Canada, Kentville, Nova Scotia, Canada

ACPEAgriculture and Agri-Food Canada, Charlottetown, Prince Edward Island, Canada

CBUCape Breton University, Sydney, Nova Scotia, Canada

CGMC	Christopher G. Majka Collection, Halifax, Nova Scotia, Canada

CNCCanadian National Collection of Insects, Arachnids, and Nematodes, Ottawa, Ontario, Canada

DHWCDavid H. Webster Collection, Kentville, Nova Scotia, Canada

JCCJoyce Cook Collection (now at the New Brunswick Museum, Saint John, New Brunswick, Canada)

JOCJeffrey Ogden Collection, Truro, Nova Scotia, Canada

KICKent Island Collection, Bowdoin College, Brunswick, Maine, USA

NSACNova Scotia Agricultural College, Bible Hill, Nova Scotia, Canada

NSMC	Nova Scotia Museum, Halifax, Nova Scotia, Canada

NSNRNova Scotia Department of Natural Resources Insectary, Shubenacadie, Nova Scotia, Canada

RMCRichard Migneault Collection, Edmundson, New Brunswick, Canada

Abbreviations: FIT, flight intercept trap.

## Identification

An identification key to species [adapted from Young (2002) and Parsons (1975)] found in the Maritime Provinces is provided below. For more detail, elytral patterns, illustrations of antennae, and general species descriptions refer to Parsons (1975).

### A. Key to genera

**Table:** 

1	Epipleural fold of elytra concave; Litargus Erichson	Litargus tetraspilotus (Fig. 9)
–	Epipleural fold of elytra horizontal and flat	2
2	Eyes transverse, sinuate anteriorly	Mycetophagus Hellwig
–	Eyes more rounded, not sinuate anteriorly; Typhaea Curtis	Typhaea stercorea (Fig. 8)

### B. Key to species of Mycetophagus Hellwig*

**Table:** 

1	Antennae gradually widening towards apex with the last 3, 4, or 5 antennomeres before the apical one more or less serrate and slightly asymmetrical; subgenus Mycetophagus (s. str.)	2
–	Antennae with a 4- or 5-segmented club, strongly to feebly differentiated from preceding antennomeres; antennomeres bilaterally symmetrical	4
2(1)	Apical antennomere longer than 2 preceding combined; length 4.6–6.3 mm	Mycetophagus punctatus (Fig. 3)
–	Apical antennomere shorter than or as long as 2 preceding combined; length 3.6 mm or less	3
3(2)	Pale elytral markings reaching or crossing suture from basal 1/5 to 1/2 of elytra	Mycetophagus flexuosus (Fig. 4)
–	Pale elytral markings not attaining suture	Mycetophagus serrulatus (Fig. 5)
4(1)	Antennae with a 5-segmented club; subgenus Ilendus Casey; length 3.2–4.7 mm	Mycetophagus pluripunctatus (Fig. 6)
–	Antennae with a 4-segmented club; subgenus Parilendus Casey; length 3.3–4.0 mm	Mycetophagus quadriguttatus (Fig. 7)

* Note: elytral markings on Mycetophagus species are variable.

## Results

In the course of this survey 175 specimens of Mycetophagidae were examined – 8 from New Brunswick, 149 from Nova Scotia, and 18 from Prince Edward Island. Included were specimens of seven species in three genera. Mycetophagus flexuosus Say is newly recorded in the Maritime Provinces from New Brunswick; Mycetophagus punctatus Say is newly recorded in the Maritime Provinces from Nova Scotia; Mycetophagus serrulatus Casey is newly recorded in New Brunswick; Mycetophagus pluripunctatus LeConte is newly recorded in New Brunswick; Mycetophagus quadriguttatus Müller is newly recorded in Nova Scotia; and Litargus tetraspilotus LeConte is newly recorded in Prince Edward Island – a total of five new provincial records, two of which are newly recorded in the region. Four species are known from New Brunswick, six from Nova Scotia, and two from Prince Edward Island (Table 1).

**Table 1.** Mycetophagidae fauna of the Maritime Provinces of Canada

		NB	NS	PE	Distribution in NE North America
Mycetophaginae					
	Mycetophagus Hellwig				
	subgenus Mycetophagus Hellwig				
Mycetophagus flexuosus Say		1			MA, ME, NB, NH, NY, ON, QC, VT
Mycetophagus punctatus Say			1		CT, MA, ME, NH, NS, NY, ON, QC, VT
Mycetophagus serrulatus Casey		1	1		NB, NH, NS, NY, ON, QC, VT
	subgenus Ilendus Casey				
Mycetophagus pluripunctatus LeConte		1	1		MA, ME, NB, NH, NS, NY, ON, QC, VT
	subgenus Parilendus Casey				
Mycetophagus quadriguttatus Müller *		1	1		MA, ME, NB, NH, NS, NY, ON, QC, VT
Typhaea stercorea (Linnaeus) †		1	1	1	MA, ME, NB, NH, NS, NY, ON, PE, QC, RI, VT
Litargus tetraspilotus LeConte			1	1	MA, ME, NH, NS, NY, ON, PE, QC, RI, VT
	totals	4	6	2	

Notes: * Holarctic species; **†** adventive Palaearctic species; **NB** New Brunswick; **PE** Prince Edward Island; **NS** Nova Scotia.

Distribution in northeastern North America: for the purposes of this treatment, northeastern North America is taken to consist of the following jurisdictions: **CT** Connecticut; **LB** Labrador; **MA** Massachusetts; **ME** Maine; **NB** New Brunswick; **NF** insular Newfoundland; **NH** New Hampshire; **NS** Nova Scotia; **NY** New York; **ON** Ontario; **PE** Prince Edward Island; **PM** Saint-Pierre et Miquelon; **QC** Québec; **RI** Rhode Island; and **VT** Vermont.

**Figure 1.**
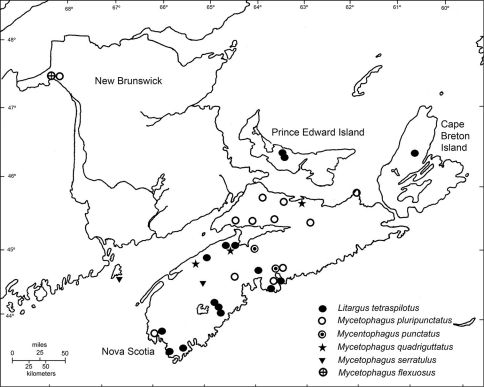
Distribution of Litargus tetraspilotus, Mycetophagus pluripunctatus, Mycetophagus punctatus, Mycetophagus quadriguttatus, Mycetophagus serrulatus, and Mycetophagus flexuosus in the Maritime Provinces of Canada.

**Figure 2.**
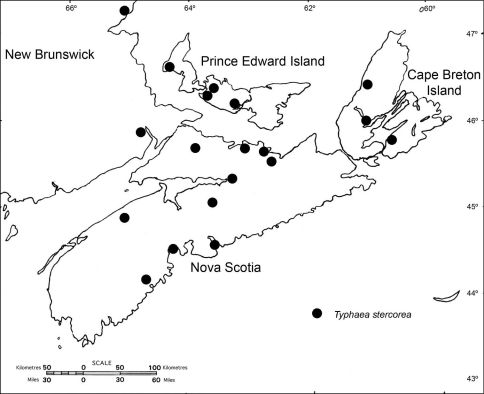
Distribution of Typhaea stercorea in the Maritime Provinces of Canada.

**Figure 3.**
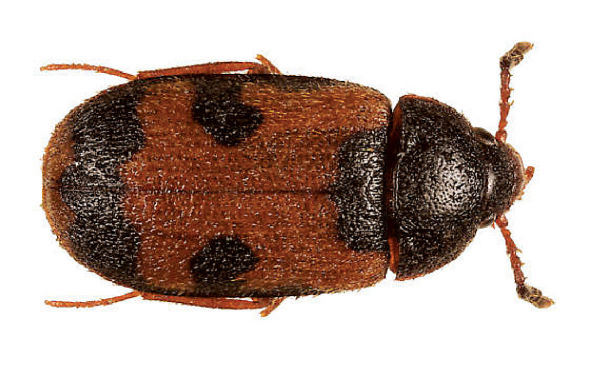
Dorsal habitus photograph of Mycetophagus punctatus. Length: 4.6–6.3 mm. Photo credit: Guy A. Hanley.

### Mycetophagus (s. str.) flexuosus

Say, 1826

#### Distribution.

**NEW BRUNSWICK: Madawaska County:** Edmundston, 47°22.285'N; 68°14.663'W, 14 August 2010, R. Migneault, in polypore on dead aspen log (1, RMC); Edmundston, 47°22.285'N; 68°14.663'W, 22 August 2010, R. Migneault, in polypore on dead aspen log (1, RMC).

#### Notes.

Mycetophagus flexuosus is newly recorded in the Maritime Provinces from New Brunswick (Fig. 1). Cline and Leshen (2005) recorded it from oyster mushroom (Pleurotus ostreatus) Fries; Weiss (1920) recorded it from turkey-tail polypore (Tramates versicolor (Fr.) Pil.); and Minch (1952) and Pielou and Pielou (1968) recorded it from birch polypore (Piptoporus betulinus) (Fr.) Kar.

**Figure 4.**
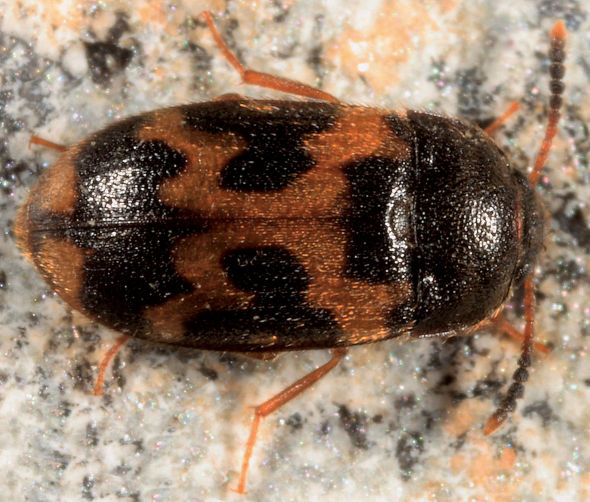
Dorsal habitus photograph of Mycetophagus flexuosus. Length: 3.0–4.6 mm. Photo credit: Tom Murray.

### Mycetophagus (s. str.) punctatus

Say, 1826

#### Distribution.

**NOVA SCOTIA: Halifax Co.:** Soldier Lake, 7 June 2005, J. Ogden, spruce beetle trap (1, NSNR); **Hants Co.:** Smileys Park, 6 July 2005, J. Ogden, spruce beetle trap (1, NSNR).

#### Notes.

Mycetophagus punctatus Say is newly recorded in the Maritime Provinces from Nova Scotia. Both specimens were collected in the central mainland of Nova Scotia (Fig. 1). The species is common under loose bark and on fungi (Downie and Arnett 1996); specifically it has been found on a dead black oak (Quercus velutina Lamb.) in Virginia (Robinson 1918); on rooting polypore (Polyporus radicatus Schw.) in Iowa (Weiss 1924); on oyster mushroom (Pleurotus ostreatus) (Cline and Leshen 2005); and on birch polypore (Piptoporus betulinus) growing on gray birch (Betula populifolia Marshall) in New York (Minch 1952).

### Mycetophagus (s. str.) serrulatus

Casey, 1900

#### Distribution.

**NEW BRUNSWICK: Charlotte Co.:** Grand Manan archipelago, Kent Island, 23 July 2012, M. Steck, balsam fir forest, sweeping (1, KIC). **NOVA SCOTIA: Annapolis Co.:** Durland Lake, 21 June 2003, P. Dollin, hemlock/balsam fir/black spruce forest (120+ years), bracket fungi on white birch (1, NSMC).

#### Notes.

Mycetophagus serrulatus Casey is newly recorded in New Brunswick. The species was reported from Nova Scotia by Dollin et al. (2008) (Fig. 1). Both specimens were found in coniferous forests, one on a polypore fungus growing on a white birch (Betula papyrifera Marshall). Cline and Leshen (2005) recorded it from oyster mushroom (Pleurotus ostreatus).

**Figure 5.**
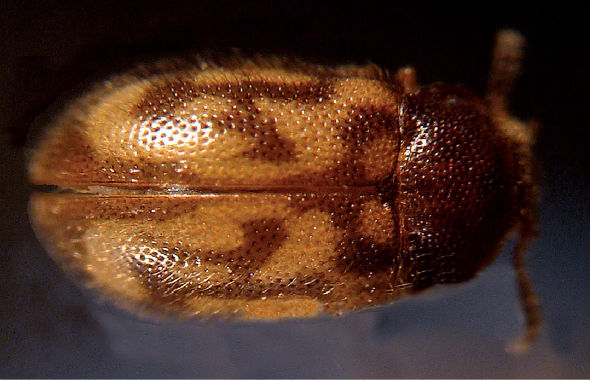
Dorsal habitus photograph of Mycetophagus serrulatus. Length: 1.3–3.6 mm. Photo credit: Christopher G. Majka.

### Mycetophagus (Ilendus) pluripunctatus

LeConte, 1856

#### Distribution.

**NEW BRUNSWICK: Madawaska County:** Edmundston, 47°22.285'N; 68°14.663'W, 22 August 2010, R. Migneault, in polypore on dead aspen log (1, RMC). **NOVA SCOTIA: Antigonish Co.:** Cape George Point, 23 June1993, M. LeBlanc, funnel trap (1, NSMC); Colchester Co.: Kemptown, 1 June 1995, 28 June 1995, C. Corkum, young deciduous forest, FIT (2, NSMC); Upper Bass River, 18 May 1995, C. Corkum, old deciduous forest, FIT (1, NSMC); Upper Bass River, 3 June 1995, C. Corkum, old deciduous forest, FIT (1, NSMC); **Cumberland Co.:** East Leicester, 2 June 1995, C. Corkum, old deciduous forest, FIT (1, NSMC); East Leicester, 14 June 1995, C. Corkum, old deciduous forest, FIT (1, NSMC); East Leicester, 15 June 1995, C. Corkum, old deciduous forest, FIT (1, NSMC); Fox River, 17 May 1995, C. Corkum, young deciduous forest, FIT (1, NSMC); Fox River, 3 June 1995, C. Corkum, young deciduous forest, FIT (1, NSMC); Harrington River, 13 July 1995, C. Corkum, young deciduous forest, FIT (1, NSMC); Wentworth, 21 May-5 July 1965, B. Wright, sugar maple forest, window trap (1, NSMC); **Halifax Co.:** Halifax, 1 December 1986, B. Wright (1, NSMC); Soldier Lake, 30 July 2004, D. MacDonald, spruce beetle trap (1, NSNR); **Lunenburg Co.:** Card Lake, 2-15 June, 1997, D.J. Bishop, red spruce/hemlock forest (old growth), FIT (1, NSMC); **Yarmouth Co.:** Wellington, 23-29 August 1992, J. & F. Cook, mixed forest (1, JCC).

#### Notes.

Mycetophagus pluripunctatus LeConte is newly recorded in New Brunswick. The species was reported from Nova Scotia by Bishop et al. (2009) and appears to be distributed throughout much of the mainland of Nova Scotia (Fig. 1). In Nova Scotia, it was collected almost exclusively with flight intercept traps in deciduous forests. Pielou and Pielou (1968) reported it on birch polypore (Piptoporus betulinus), Cline and Leshen (2005) recorded it from oyster mushroom (Pleurotus ostreatus), and Leschen (1988) recorded it from Spongipellis unicolor (Schw.) growing on a fallen white oak (Quercus alba L.) in Arkansas. Schwartz (1876) said it was “abundant in fungus” in Michigan.

**Figure 6.**
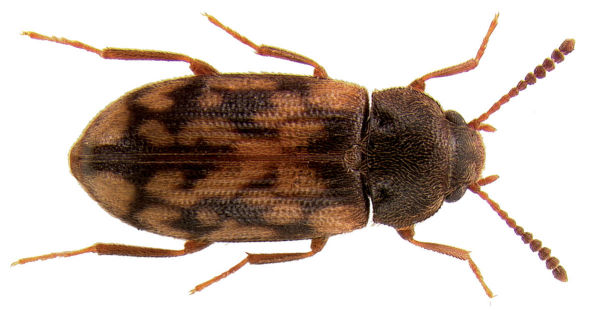
Dorsal habitus photograph of Mycetophagus pluripunctatus. Length 3.2–4.7 mm. Photo credit: Nicholas Gompel.

### Mycetophagus (Parilendus) quadriguttatus

Müller, 1821

#### Distribution.

**NOVA SCOTIA: Annapolis Co.:** Paradise, 11 June 2005, K. Webster, spruce beetle trap (1, NSNR); **Colchester Co.:** Balmoral Mills, 19 June 1974, B. Wright, grist mill (1, NSMC); **Kings Co.:** Kentville, 10 August 2005, D.H. Webster, compost heap, moldy corncobs (1, DHWC).

#### Notes.

Mycetophagus quadriguttatus Müller is newly recorded in Nova Scotia (Fig. 1). The species was reported from New Brunswick by Bousquet (1991), however, I have not been able to locate a voucher specimen for this record; it is not present in the CNC nor was it reported from New Brunswick by Campbell et al. (1989). Pending confirmation its status in New Brunswick should be regarded as provisional. In Nova Scotia, one specimen was collected in a grist mill and another in a compost heap. Campbell et al. (1989) reported the species in waste feed, sacked grain, grain elevators, warehouses, flour mills, old flour barrels, fungi at the base of old hay stacks, fungi on trees, a vegetable store, and a corn shop.

Although Hatch (1962) thought it was probably an introduced species, other investigators (Parsons 1975; Bousquet 1991; Downie and Arnett 1996) have classified it as a native Holarctic species. Mycetophagus quadriguttatus is widely distributed in Europe having been reported throughout the continent except for Corsica, Crete, Cyprus, Estonia, Ireland, Norway, Portugal, and Sicily (Nikitsky 2010), and is also found across North Africa, in the eastern Palaearctic, Asia, and Australia (Nikitsky 2010).

**Figure 7.**
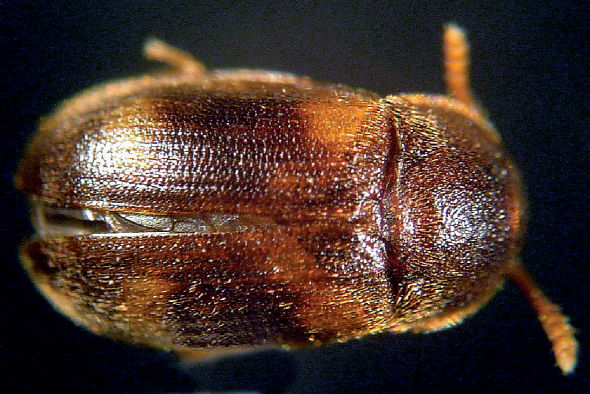
Dorsal habitus photograph of Mycetophagus quadriguttatus. Length 3.3–4.0 mm. Photo credit: Christopher G. Majka.

### Typhaea stercorea

(Linnaeus, 1758)

#### Distribution.

Eighty-two specimens (NB=6, NS=66, PE=12) were examined. The earliest records from each province are: **NEW BRUNSWICK: Northumberland Co.:** Tabusintac, 13 June 1939, 26 July 1939, W.J. Brown (2, CNC). **NOVA SCOTIA: Colchester Co.:** Truro, 4 March 1919, collector not recorded (8, NSAC). **PRINCE EDWARD ISLAND: Prince Co.:** Central Bedeque, 29 July 1954, F.M. Cannon (1, ACPE).

#### Notes.

Typhaea stercorea (Linnaeus) was reported from New Brunswick, Nova Scotia, and Prince Edward Island by Bousquet (1991). The species is widely distributed throughout the Maritime Provinces, including Cape Breton Island (Fig. 2). A majority of specimens were collected outdoors in native habitats. It is an adventive Palaearctic beetle found both outdoors and in association with various stored products. Typhaea stercorea has been found in corn fields (on decaying kernels of exposed ears), warehouses, stores, flour mills, mangers, railway boxcars, dwellings, and granaries in stored grain and seeds, tobacco, peanuts, cacao, corn, millet, wheat, apricots, and moldy grape skins, as well as in nests of swans and moorhens (Campbell et al. 1989). In Nova Scotia it was reported in large numbers in dairy barns (Campbell et al. 1989).

The dates of earliest detection are given above: New Brunswick (1939), Nova Scotia (1919), and Prince Edward Island (1954). Typhaea stercorea is widespread in Europe, having been recorded in every country and region in the continent (Nikitsky 2010), and is also virtually cosmopolitan globally, being found in every region of the world except (doubtfully) South and Central America (Nikitsky 2010).

**Figure 8.**
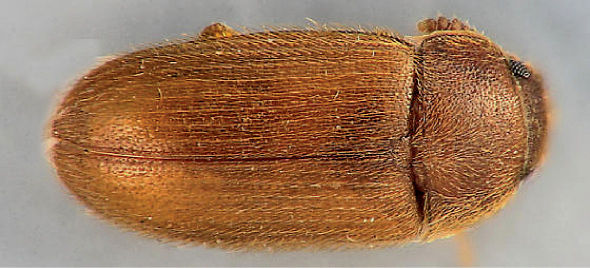
Dorsal habitus photograph of Typhaea stercorea. Length: 2.2–3.2 mm. Photo credit: Tim Moyer.

### Litargus tetraspilotus

LeConte, 1856

#### Distribution.

**NOVA SCOTIA: Cape Breton Co.:** East Bay, 9 September 2003, C.W. D’Orsay (1, CBU); **Colchester Co.:** Bible Hill, 8 July 2004, K.R. Aikens, pasture, sweep (1, CBU); Bible Hill, 14 June 2005, S.M. Townsend, sweep (3, CBU); Debert, 9 June 1994, J. Ogden (1, NSNR); Masstown, 7 September 2002, C.G. Majka, marshy swamp (1, CGMC); Shubenacadie, 26 August 1997, J. Ogden (1, NSNR); **Digby Co.:** Brier Island, Pond Cove, 9 August 2004, J. Ogden & K. Goodwin, knapweed, sweep (3, JOC); Brier Island, Pond Cove, 10 August 2004, J. Ogden & K. Goodwin, sweep (1, JOC); Brier Island, Westport, 9 August 2004, J. Ogden & K. Goodwin, grassland, sweep (2, JOC); **Halifax Co.:** Big Indian Lake, 16 July 2003, P. Dollin, Picea rubens forest (80-120 years), in rotting mushroom (1, NSMC); Point Pleasant Park, 15 August 2000, 7 September 2000, C.G. Majka, mixed forest (2, CGMC); Point Pleasant Park, 9 September 2000, 2 June 2002, 23 July 2002, C.G. Majka, coniferous forest (3, CGMC); Point Pleasant Park, 12 May 2001, 10 June 2001, 25 May 2002, C.G. Majka, coniferous forest, on Picea rubens (6, CGMC); Point Pleasant Park, 19 May 2001, 29 May 2001, C.G. Majka, coniferous forest, on Pinus strobus (4, CGMC); 29 July 2001, 18 August 2001, Point Pleasant Park, C.G. Majka, mixed forest (2, CGMC); Point Pleasant Park, 9 May 2002, C.G. Majka, coniferous forest, on Abies balsamea (1, CGMC); Point Pleasant Park, 9 June 2002, C.G. Majka, mixed forest, on Aralia hispida (1, CGMC); Point Pleasant Park, 7 July 2002, C.G. Majka, seashore (1, CGMC); Point Pleasant Park, 14 September 2002, C.G. Majka, marsh, on herbaceous vegetation (1, CGMC); Point Pleasant Park, 30 June 2004, C.G. Majka, coniferous forest, on Pinus sylvestris (2, CGMC); West Dover, 7 September 2003, C.G. Majka, coastal barrens, heaths (1, CGMC); **Kings Co.:** Aldershot, 5 August 1949, 2 August 1949, 10 August 1949, 20 August 1949, 16 May 1950, H.T. Stultz (5, ACNS); Greenwich, 29 May 1958, H.T. Stultz (1, ACNS); Kingston, 30 June 2002, C.G. Majka, sandy pine barren (1, CGMC); **Queens Co.:** Eight Mile Lake, 11 August 2003, P. Dollin, Picea rubens forest (40-80 years), in vegetation, sweep (1, NSMC); Little Ponhook Lake, 1 August 1993, B. Wright, in oak apple galls (3, NSMC); Ponhook Lake nr. Greenfield, 13 July 1993, J. Cook, ultraviolet light trap (2, JCC); **Shelburne Co.:** Clyde River Road, 16 July 1992, S. & J. Peck, forest, car net (1, JCC); Forbes Point, 9 July 2007, R. Gorham, grass/alders (4, CGMC); **Victoria Co.:** Cape Breton Highlands: Kelly Rd, 24 June 2005, J. Ogden, malaise trap (1, NSNR); **Yarmouth Co.:** Moses Lake, 8 km N of Argyle, 17-22 July 1993, J. & T. Cook, mixed forest, FIT (1, JCC). **PRINCE EDWARD ISLAND: Queens Co.:** Cavendish, 19 July 2001, C.G. Majka, coastal vegetation (1, CGMC); Princeton-Wharburton Road, 19 August 2002, C.G. Majka, old field (3, CGMC); St. Patricks, 18 August 2002, C.G. Majka, old field (1, CGMC); St. Patricks, 29 June 2003, C.G. Majka, mixed forest (1, CGMC).

#### Notes.

Litargus tetraspilotus LeConte is newly recorded from Prince Edward Island. Klimaszewski and Majka (2007) first reported this species in Nova Scotia. There are many records from the southern mainland of Nova Scotia, Cape Breton Island, and Prince Edward Island (Fig. 1). Records from New Brunswick and the northern mainland of Nova Scotia are lacking, but it is probable that it is found throughout the region.

In the Maritime Provinces Litargus tetraspilotus has been collected in many habitats including coniferous, deciduous, and mixed forests, seashores, coastal barrens, grasslands, marshy areas, a sandy pine barren, and an old field ecosystem. Specimens have been collected on the foliage of white pine (Pinus strobus L.), jack pine (Pinus sylvestris L.), red spruce (Picea rubens Sarg.), balsam fir (Abies balsamea (L.) Mill.), on deciduous, and herbaceous vegetation, on bristly sarsaparilla (Aralia hispida Vent.), and in a rotting mushroom. Klimaszewski and Majka (2007) reported Litargus tetraspilotus as an inquline inhabitant of oak apple galls on red oak (Quercus rubra L.) induced by Andricus (Callirhytis) sp. (Cynipidae) wasps. Rauf et al. (1985) found it on jack pine, Tucker (1919) found it on American mistletoe (Phoradendron flavescens (Pursh) Nutt., and Ulyschen and Hanula (2010) reared it from decomposing loblolly pine (Pinus taeda L.) logs in South Carolina.

**Figure 9.**
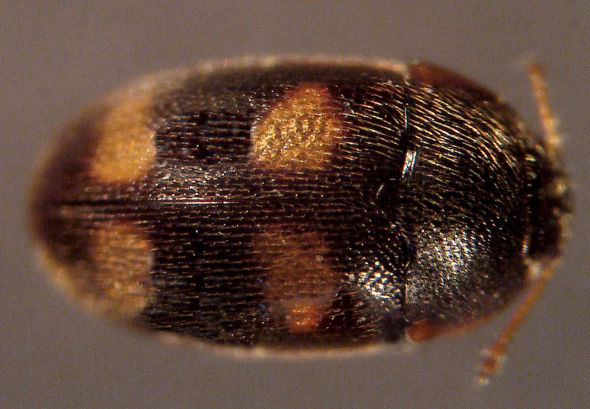
Dorsal habitus photograph of Litargus tetraspilotus. Length: 1.8–2.0 mm. Photo credit: Christopher G. Majka.

## Discussion

Typhaea stercorea and Litargus tetraspilotus are abundant and widely distributed in the Maritime Provinces. Mycetophagus pluripunctatus appears to be uncommon but widely distributed on the mainland of Nova Scotia. The other four species of mycetophagids – Mycetophagus punctatus, Mycetophagus flexuosus, Mycetophagus serrulatus, and Mycetophagus quadriguttatus – are all represented by a handful of specimens or less. They would all appear to qualify as “apparently rare” saproxylic beetles as defined by Majka (2007b) (i.e., representing < 0.005% of specimens examined from the region). In investigating 283 species of saproxylic beetles from 18 families, Majka (2007b) found that 33% of these fell into this category of apparently rare species. Similarly in examining the Endomychidae and Erotylidae of the Maritime Provinces, two other families of beetles closely associated with fungi, Majka (2007b) found that 40% of the 15 species found in the region are apparently rare. Majka (2007a, b) suggested that this large proportion might be ascribable to the history of forest management practices in the region. These apparently rare species of Mycetophagus, three of which are closely associated with saproxylic fungi, may belong to this same suite of insects for similar reasons.

In general, mycetophagids have received rather little attention by researchers in North America, and the bionomics of many species have not been carefully investigated. Certainly this is true in the Maritime Provinces and additional fieldwork in the region is required to ascertain more about their distribution, abundance, bionomics, and ecological role in the habitats that they inhabit.

## Supplementary Material

XML Treatment for Mycetophagus (s. str.) flexuosus

XML Treatment for Mycetophagus (s. str.) punctatus

XML Treatment for Mycetophagus (s. str.) serrulatus

XML Treatment for Mycetophagus (Ilendus) pluripunctatus

XML Treatment for Mycetophagus (Parilendus) quadriguttatus

XML Treatment for Typhaea stercorea

XML Treatment for Litargus tetraspilotus
